# Thiol Modifications in the Extracellular Space—Key Proteins in Inflammation and Viral Infection

**DOI:** 10.3389/fimmu.2022.932525

**Published:** 2022-06-27

**Authors:** Kathrin A. Brücksken, Paola Loreto Palacio, Eva-Maria Hanschmann

**Affiliations:** Department of Neurology, Medical Faculty, Heinrich-Heine University, Düsseldorf, Germany

**Keywords:** thiol switch, disulfide bond, redox signaling, extracellular, inflammation, infection, S-glutathionylation, S-nitrosylation

## Abstract

Posttranslational modifications (PTMs) allow to control molecular and cellular functions in response to specific signals and changes in the microenvironment of cells. They regulate structure, localization, stability, and function of proteins in a spatial and temporal manner. Among them, specific thiol modifications of cysteine (Cys) residues facilitate rapid signal transduction. In fact, Cys is unique because it contains the highly reactive thiol group that can undergo different reversible and irreversible modifications. Upon inflammation and changes in the cellular microenvironment, many extracellular soluble and membrane proteins undergo thiol modifications, particularly dithiol–disulfide exchange, S-glutathionylation, and S-nitrosylation. Among others, these thiol switches are essential for inflammatory signaling, regulation of gene expression, cytokine release, immunoglobulin function and isoform variation, and antigen presentation. Interestingly, also the redox state of bacterial and viral proteins depends on host cell-mediated redox reactions that are critical for invasion and infection. Here, we highlight mechanistic thiol switches in inflammatory pathways and infections including cholera, diphtheria, hepatitis, human immunodeficiency virus (HIV), influenza, and coronavirus disease 2019 (COVID-19).

## Introduction

Following transcription and translation, synthesized proteins can undergo posttranslational modifications (PTMs). These constitute regulatory mechanisms to control molecular and cellular functions in response to signals and changes in the microenvironment. Astonishingly, 300–400 different modifications have been described, including catalytic cleavage, phosphorylation, glucosylation, ubiquitinylation, methylation, and oxidation (1, 2). The spatiotemporal and the interconnectivity analysis of different PTMs is demanding, even though biochemical methods based, e.g., on specific antibodies, large-scale experimental tools such as mass spectrometry (3–5), and computational methods including the analysis of putative modifications are available (1, 2). Particularly, the analysis of PTMs in the extracellular space is challenging. However, it is clear that PTMs, e.g., i) of the extracellular matrix (ECM) play an essential role in signal transduction (6) and ii) are involved in extracellular vesicle (EV) biogenesis, cargo sorting, and vesicular uptake (7, 8).

### Posttranslational Thiol Modifications

The highly reactive thiol group of cysteines (Cys) is special because it can undergo many different reversible and irreversible modifications that control protein activity, interaction, and/or distribution (9). Single Cys modifications include S-glutathionylation [addition of glutathione (GSH)] and S-nitrosylation [incorporation of nitric oxide (NO)]. Moreover, two Cys residues can be oxidized and form a disulfide bond that has additionally been linked to protein structure and conformation. As such, they depend on the presence of enzymatically produced second messengers like hydrogen peroxide (H_2_O_2_) and NO and the catalysis by Thioredoxin (Trx) family proteins such as Trx, Glutaredoxins (Grx), Peroxiredoxins (Prx), and Protein disulfide isomerases (PDIs) (10). All these modifications are important not only for intracellular but also for extracellular signal transduction (i.e., oxidative eustress), also for regulating membrane, soluble, and vesicular proteins (11, 12).

## Thiol Modification of Extracellular Proteins in Inflammatory Signaling

Thiol modifications are essential in the innate and adaptive immune response, controlling various pathways and functions, by distinct catalytical mechanisms in different cellular and extracellular compartments and specialized cell types (13). Here, we focus on extracellular dithiol–disulfide exchange, S-glutathionylation, and S-nitrosylation of key proteins in inflammation and infection, as well as the redox proteins involved in the regulation of these PTMs (**Table 1**).

**Table 1:** Posttranslational thiol modifications of proteins involved in inflammation and infection.

Protein	Function	Modification	Regulation	Reference
**Alpha 1 antitrypsin**	Glycoprotein; mainly produced by hepatocytes	S-Nitrosylation: Increases bacteriostatic function and activation of immune cells	Unknown	(14)
**Beta-1 defensin**	Interaction with microbial targets; antimicrobial peptide	Reduced: Increases antimicrobial and chemotactic activity Disulfide: Facilitates stability of a compact, highly ordered fold	Catalysis by Trx1	(15, 16)
**Cathepsin B**	Protease; involved in various physiological processes	S-Glutathionylation leads to stability without affecting enzyme activity	Unknown	(17–19)
**Cluster of differentiation 4**	Transmembrane glycoprotein; acts as a coligand and coreceptor of MHC II molecule	Reduced: Improves affinity toward the T-cell receptor; HIV-1 entry Disulfide: Reduces the affinity for cell entry (HIV-1)	Catalysis by Trx1	(20, 21)
**Cyclooxygenase-2**	Key enzyme required for the conversion of arachidonic acid into prostaglandins	S-Nitrosylation: Activates eicosanoid production	Catalysis by Grx1	(22, 23)
**Diphtheria toxin**	Toxin synthesized by *Corynebacterium diphtheriae*	Reduced disulfides generate two fragments; induce translocation Disulfide: Activates ADP-ribosyltransferase activity and eventually inhibits protein biosynthesis	Catalysis by Trx1	(24–26)
**Glycoprotein 120**	Interaction with viral coreceptors present on the lymphocyte surface	Disulfide: Reduces the affinity for viral attachment to the host lymphocyte cell surface and invasion	Trx1: Indirectly regulates Gp120 binding to CD4	(27, 28)
**High-mobility group box 1**	Nuclear DNA-binding protein; functions as a pro-inflammatory cytokine	Disulfide bridges and S-Glutathionylation essential for binding of TLRs and RAGEs	Oxidation by Prx1/Prx2 Interaction with Grx1	(29, 30) (29)
**Heat shock protein 60**	Chaperone	S-Nitrosylation: Regulates mitochondrial DNA stability and protein binding	Catalysis by Trx1	(31, 32)
**IgG**	Immunoglobin; antibody; recognition and binding of antigens	Reduced: Increases the antigen affinity Disulfide bridges lead to the stability of the antibody and isoform formation	Catalysis by Trx1	(33, 34)
**Integrin**	Regulation of cellular growth, proliferation, migration, signaling, and cytokine activation and release	S-Glutathionylation of α4 enhances affinity for neutrophil Vascular cell adhesion protein (VCAM) and mobilization of cells out of the bone marrow Disulfide of α7β1 affects integrin-mediated cell adhesion	Catalysis by Grx1 Catalysis by Trx1	(35) (36)
**Interleukin-1β**	Cytokine	S-Glutathionylation: Regulates activity	Catalysis by Grx1	(37)
**Signal transducer and activator of transcription 1**	Transcription factor; regulation of inflammatory responses and cellular death	S-Glutathionylation: Increases activity and induces CXC9 in macrophages	Unknown	(38, 39)

### Dithiol–Disulfide Exchange

Disulfide formation occurs between two thiols of one (intramolecular) or two (intermolecular) proteins. Conformational disulfides are important for the structure and function of specific proteins and their regulation. Several intracellular thiol switches were identified that involve the regulated formation or reduction of disulfide bonds, e.g., Collapsin Response Mediator Protein 2 (Cys504-Cys504) (40, 41), Glyceraldehyde 3-phosphate dehydrogenase (GAPDH) (42), Heat shock protein (HSP) 33 (Cys232–Cys234) (43, 44), H_2_O_2_-inducible gene activator (Cys199-Cys208) (45, 46), and yes-associated protein 1 (Cys303-Cys598) (47). Interestingly, the extracellular space was considered to be rather oxidizing and disulfides inert, not engaging in redox regulation. However, it is an exciting time when this view is challenged. Redox regulation of proteins not only occurs extracellularly but also is in fact an important part of various signaling cascades. Even though the function of many proteins was shown to be sensitive toward oxidation by H_2_O_2_, not many extracellular thiol switches have been identified and thoroughly characterized so far. Known examples were described in recent review articles (12, 48). A well-characterized soluble protein is High-mobility group box 1 (HMGB1). In its disulfide form (Cys23-Cys45), HMGB1 binds to Toll-like receptors (TLRs) and the receptor for advanced glycation end products (RAGE), inducing the signal cascade that leads to Nuclear factor kappa B (NFκB) activation and cytokine expression and release (49, 50). The interaction of HMGB1 with TLRs also depends on the redox state of the third Cys residue (51). Dithiol–disulfide exchange in membrane proteins such as A disintegrin and metalloprotease 17 (ADAM17) (Cys600-Cys635; Cys630-Cys641) (52, 53), cluster of differentiation 4 (CD4) (intermolecular, Cys130-Cys159) (20), beta-1 defensin (15), various integrins (36, 54–56), tumor necrosis factor receptor superfamily member 8 (CD30) (57), and transient receptor potential canonical channels (58, 59) affects ligand binding, protein function, and cell signaling. Beta-1 defensin increases its antimicrobial activity when reduced. Inhibition and knockout of Trx decreased the rate of Beta-1 defensin degradation, directly affecting its antimicrobial function. CD4, CD30, and α7β1-integrin are also substrates for extracellular Trx (36, 60). ADAM17 is regulated by PDIs (52).

Conformational disulfides are particularly important in the adaptive immunity, as they are essential for the structure and function of antibodies and antigen presentation. PDIs have been associated with increased production of immunoglobulin heavy chains (61), including most of the IgG isoforms. *In vitro*, PDIs are able to replicate disulfide bonds in antibody formation as it occurs *in vivo*, and this is even more upon oxidizing conditions (62). PDIs are involved in the modification of immunoglobulins, including IgG (63) and IgM (64). The IgM pentameric structure is the first antibody produced by B-cells before undergoing class switching. PDIs regulate the biogenesis of this multimeric immunoglobulin by catalyzing the formation of disulfide bonds, structures that are later modified before secretion (64). IgG is vital for immunological memory. Several isoforms have been described with differences in their constant regions (63). Disulfide bonds vary across the chains in different IgG isoforms, changing the conformation and subsequently antibody stability and antigen affinity (33). Remarkably, all IgG isoforms can also present non-classical disulfide conformations, such as trisulfides (65). One of the IgG isoforms most studied for its non-classical conformation is IgG4. A change in amino acid in the hinge region makes it different from IgG1 and more prone to form intrachain bonds, rather than interchain bonds. This change in IgG4 has been hypothesized to be key in its role as a monovalent antibody (66) and has been associated with protective effects after prolonged immunizations (67). Interestingly, its production does not seem to be affected by PDI expression (68). Oxidation of immunoglobulins is a problem that has been studied particularly *in vitro*, especially concerning protein storage for medical treatments or experimental purposes. Environmental oxidizing conditions lead to changes in amino acid charges and specificity of the antibody for its antigen (69). Researchers have been actively working on solving this problem in the production and distribution of immunoglobulins by using Trx1 (70), Trx-like proteins (71), and a combination of GSH and Trx1 (72). Not many *in vivo* experiments have shown the impact of redox reactions on antibody function. However, it has been shown that the redox environment could affect the functionality of antibodies and potentially disease diagnosis (73). *In vivo*, modifications to the heavy-chain disulfide bonds of, e.g., IgG1 subclass proteins alter the binding site of the C1 complement protein (74). This interaction is key for the complement classical pathway activation. We can hypothesize that these changes are constantly occurring *in vivo*, but how this is affecting and modulating the immune response remains to be elucidated. Oxidation of the immunoglobulins might not be the only factor affecting the adaptive immune response. Peptides presented to T-cell receptors *via* major histocompatibility complex (MHC) class II can be modified if they contain Cys residues (75, 76). Changes in antigen recognition can change when these residues form disulfide structures with peptides in the vicinity (75), or are S-glutathionylated, which modulates the immune response against viruses and T cell-mediated tumor cell recognition (76). Future research is needed to determine if these changes are active mechanisms for the immune escape of infected or neoplastic cells.

### S-Glutathionylation

**Figure 1:**
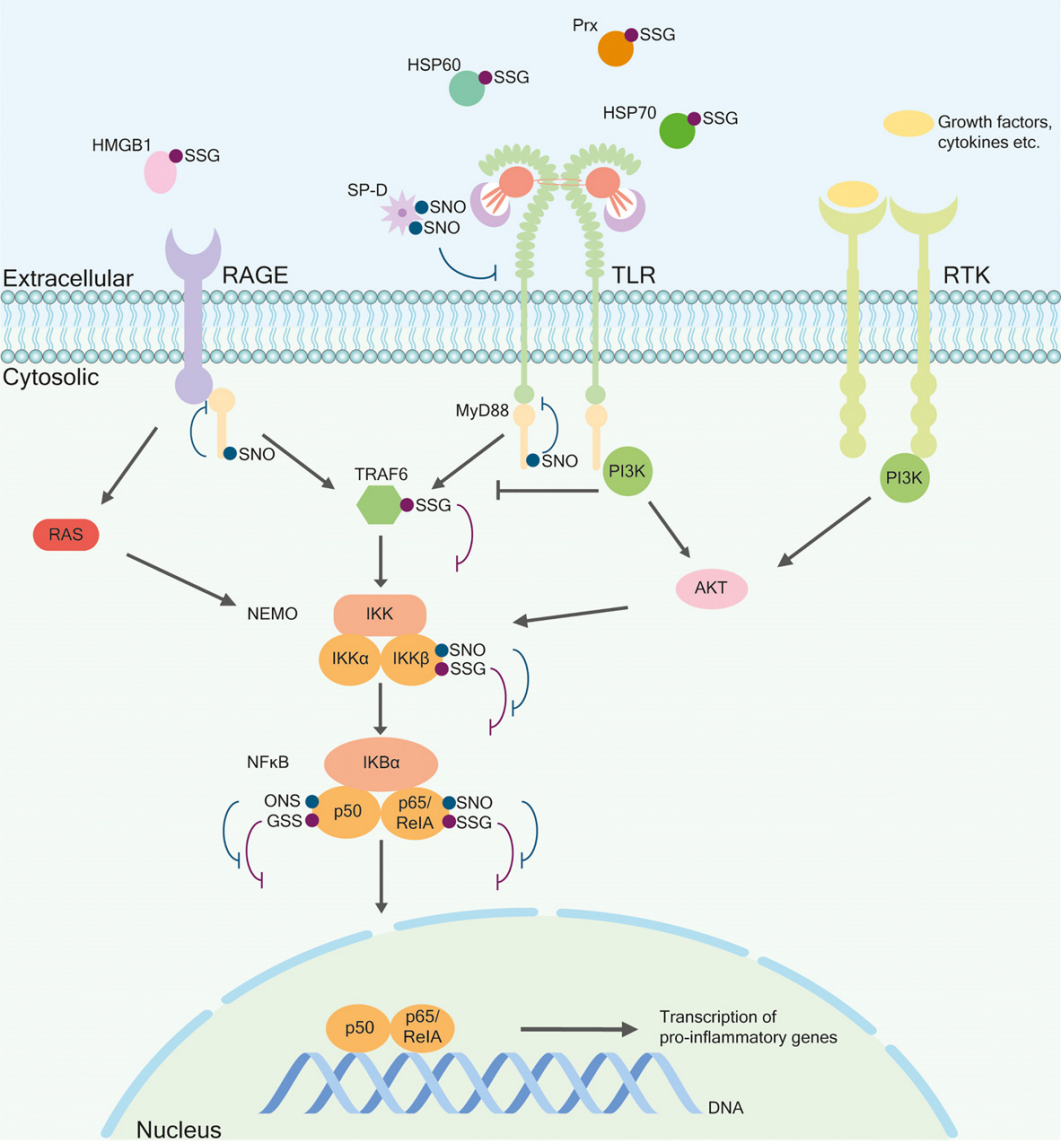
Selected thiol modifications in the regulation of the inflammatory response. Posttranslational modifications such as S-glutathionylation and S-nitrosylation occur on intracellular and extracellular proteins. Many ligands for membrane-bound receptors such as receptors for advanced glycation end products (RAGE), Toll-like receptor (TLR), and receptor tyrosine kinase (RTK) undergo redox regulation. Nitrosylated, oligomeric Surfactant protein D (SP-D) binds and inhibits TLR. Heat shock proteins (HSPs) 60 and 70, High-mobility group box 1 (HMGB1), and Peroxiredoxin (Prx) are glutathionylated. They bind to RAGE or TLR, inducing similar downstream-signaling components Tumor necrosis factor receptor–associated factor (TRAF), Nuclear Factor-kappa-B essential modulator (NEMO), Nuclear Factor-kappa-B (NFκB) and eventually a pro-inflammatory response. The RAGE receptor can also activate the NEMO complex *via* RAS. Also, the TLR pathway can be activated through the Phosphoinositide 3-kinases (PI3K) pathway. PI3K binds to the receptor, which inhibits TRAF6 and activates the serine/threonine-protein kinases (Akt) pathway by activating NEMO. Different components of this pathway can be glutathionylated or nitrosylated. Nitrosylated myeloid differentiation primary response 88 (MyD88) leads to the detaching of the receptor whereby the signal is inhibited. Nitrosylated or glutathionylated IKKβ, p50, and ReIA/p65 lead to their inactivation, eventually inhibiting gene expression.

S-glutathionylation is the formation of a disulfide bond between the thiol group of GSH and a protein (77), catalyzed by Grxs (78). Intracellular proteins that can be glutathionylated include actin (Cys374) (79), Fatty Acid Binding Protein 5 (80), Major vault protein (81), NFκB (82, 83), sarco/endoplasmic reticulum Ca^2+^-ATPase (Cys674) (84), Signal transducer and activator of transcription 1 (STAT1) (Cys324 and Cys492) (39, 85), STAT3 (Cys328 and Cys542) (85), and members of the Trx family (17, 86, 87). Glutathionylation is crucial for signaling pathways and cellular processes like proliferation, differentiation, apoptosis, cytokine production (76), and metabolic changes in response to inflammatory signals (88). Also, the release of glutathionylated proteins upon inflammation and infection has been described (17). The functional characterization of extracellular proteins that undergo glutathionylation, however, is considerably rare. Examples include several soluble proteins and receptors that function in signal transduction and inflammation such as Intercellular Adhesion Molecule 1 (89), α4β1 integrin (90), HMGB1 (29), interleukin 1 β (IL-1β) (37), and Paraoxonase-1 (91). Glutathionylation of IL-1β (Cys188) does not directly control the bioactivity under physiopathological conditions, but it protects IL-1β from H_2_O_2_-induced irreversible deactivation, which is regulated by Grx1 (37). Trx1 (Cys73) is also glutathionylated, which prevents dimerization or limited proteolysis. The latter gives rise to the truncated form Trx80. This form is released into the extracellular space and has cytokine-like activity (86, 92). Prx1 and Prx2 are glutathionylated (17, 87) and are known to act as danger signals by binding to TLR4 (93, 94) (**Figure 1**). Other ligands of TLR2 and 4 like HSPs 60 and 70 (Cys574 and Cys603) (95) can be glutathionylated in T-lymphocytes in response to inflammatory stimuli and H_2_O_2_ (96–98). Extracellular HMGB1 also binds to TLRs and RAGE, triggering the production of pro-inflammatory cytokines (**Figure 1**). Downstream signal transduction also involves glutathionylated mediators, such as IKKβ, which reduces nuclear translocation of ReIA (p65), inhibiting DNA binding. ReIA (p65) and the p50 subunit of NFκB also undergo glutathionylation, resulting in decreased ability to bind DNA (83) (**Figure 1**). Of note, the pro-inflammatory response triggered by IL-17a is also associated with glutathionylation of ReIA (p65) and IKKα (99).

### S-Nitrosylation

S-nitrosylation is the covalent binding of NO to a thiol group and regulates >3,000 proteins (100), affecting protein structure, function, and the interconnectivity with other PTMs like phosphorylation, acetylation, and disulfide formation (101–104). Identified intracellular S-nitrosylated proteins include Cofilin-1 (Cys80, Cys139) (105, 106), GAPDH (Cys150, Cys247) (107–109), HSP60 (Cys237) (31, 105), HSP90 (Cys521) (110, 111), MyD88 (Cys113, Cys216) (112, 113), NFκB (114, 115), Nitric oxide synthase (NOS) (116, 117), and calcium- and zinc-binding proteins S100A8 (Cys42) (117) and S100A9 (108). Interestingly, Trx1 and PDIs can be nitrosylated and act as transnitrosylases (32, 118). Furthermore, Trx1 catalyzes the denitrosylation of substrate proteins like HSP60 and cofilin-1 (105). S-nitrosylation is involved in various physiological and pathophysiological processes, including apoptosis, DNA damage repair, inflammation, mitochondrial energy metabolism, proliferation, and regulation of transcription (119, 120). Extracellular proteins and receptors include Aquaporin-1 (AQP-1) (121), CD40 (122), Epidermal Growth Factor Receptor (123), insulin-like growth factor type 1 receptor (124), and Surfactant Protein D (SP-D) (Cys15, Cys20) (125–127). S-nitrosylation of SP-D leads to oligomerization of the protein (125) and TLR4 binding and inhibition (126, 127) (**Figure 1**). Additional nitrosylation of SP-D (Cys15/20) separates the oligomeric form into trimers, exposing the N-terminal domain and leading to chemotaxis of macrophages (125). S-nitrosylation of MyD88 within the TLR pathway disrupts its binding to upstream Toll/IL-1R adaptor protein (TIRAP) but not to downstream Interleukin-1 receptor-associated kinase 1 (IRAK-1). This could influence the delayed development of the acute immune response (112). The subunits p65 and p50 of NFκB can become nitrosylated, which has a similar inhibitory effect on DNA binding as glutathionylation. Nitrosylation of both subunits leads to inactivation of the complex (114, 115) (**Figure 1**). In addition, IKKβ is nitrosylated at Cys179, and this PTM is reversed upon Tumor necrosis factor α activation (128). Among membrane proteins, channels can be nitrosylated such as AQP-1 at Cys189, located within the functional pore, allowing potential negative feedback regulation by inhibiting the function of AQP-1 (reviewed in 121). Nitrosylation of CD40 prohibits binding and activation by CD40L. This modification occurs in the extracellular domain in resting macrophages and monocytes. Denitrosylation occurs after activation by CD40L, resulting in the activation of the NFκB pathway (122).

## Thiol Switches in Bacterial and Viral Infections

PTMs of thiols, i.e., mainly regulatory disulfides, mediate viral entry into host cells. Specific thiol switches occur in major viral infections including hepatitis, human immunodeficiency virus (HIV), and influenza. Recent studies also imply the presence of a relevant thiol switch that facilitates the entry of SARS-CoV-2. Even though there is more evidence on viral infections, critical thiol modifications also occur in bacterial infections.

### Bacterial Infections

The Gram-positive *Corynebacterium diphtheriae* is the cause of diphtheria that affects the respiratory tract and skin. Diphtheria toxin (DT) is released as a virulence factor and enters cells *via* receptor-mediated endocytosis. It contains an intermolecular disulfide bridge (Cys186, Cys201) and depends on host cell-mediated reduction (24), which occurs on the cell surface (25), and is catalyzed by PDIs (25). *In vitro*, Trx1 reduces DT at pH 5 and GSH or Cys at neutral pH (26). Following reduction, the N-terminal part containing the ADP-ribosyltransferase activity enters the cytoplasm—a process that among others depends on Trx reductase—and catalyzes the ADP-ribosylation of elongation factor (EF) 2 and thereby inhibits protein synthesis, which eventually induces apoptosis (24). A comparable mechanism was found for the Gram-negative *Pseudomonas aeruginosa.* Released Exotoxin A contains a disulfide (Cys265, Cys287) that is cleaved by PDIs and eventually leads to inhibition of protein synthesis. Interestingly, the reduction does not occur on the cell surface, but intracellularly (129, 130). For the Gram-negative *Vibrio cholera*, a disulfide bond (Cys-187, Cys-199) in cholera toxin was identified that is also a substrate for intracellular PDIs (131–133).

Besides activating bacterial toxins, some bacterial strains were shown to rely on disulfide reductases on the cell surface for invasion and infection. The Gram-negative *Anaplasma phagocytophilum*, the cause of anaplasmosis, expresses the adhesin Asp14 required for host invasion. Asp14 binds and brings PDI proximal to the bacterial surface. The substrate disulfide bonds on the bacterial surface have not been identified; however, a reduction is needed. Interestingly, extracellularly, membrane-associated Trx1 mediates bacterial entry (134). Similarly, the Gram-negative *Ehrlichia chaffeensia*, the cause of human monocytic ehrlichiosis, expresses an adhesin EplA that binds PDIs and mediates bacterial entry into host cells. Inhibition of Trx1 prohibits infection (135).

### Viral Infections

The enveloped HIV is the cause of acquired immunodeficiency syndrome (AIDS). The viral envelope glycoprotein 120 (gp120) is rich in disulfide bonds and is essential for viral attachment to the host lymphocyte cell surface. Following binding to receptor CD4 and coreceptor CXCR4, conformational changes, i.e., the reduction of two intramolecular disulfides, allow fusion, viral entry, and infection. The reduction is catalyzed by PDIs (27, 28), Grx1 (136), and Trx1 (137). Interestingly, PDI is mainly involved in infection of T-lymphocytes and Trx1 in infection of macrophages. Trx1 is particularly elevated in chronic phases of HIV infection. The authors concluded that Trx1 may enable sustained viremia, when T-lymphocytes are declining (138). Another mechanistic thiol switch was identified for the enveloped hepatitis C virus that causes hepatitis C. The envelope glycoproteins E1 and E2 contain several conserved Cys residues. Cellular attachment is enabled by free thiol groups in E1 and E2 and not by disulfide formation (139). Mutagenesis screenings of E1 (140) and E2 (141) revealed the contributions of Cys residues on structure and function. All 18 Cys residues of E2 are needed for viral entry, even though the contribution to structure and function is different (141). Thiol switches in other viral strains have been identified and analyzed (142), including the Hepatitis A Virus-2B (HAV-2B) peptide (142), vaccinia virus proteins (143), and adenoviral capsid protein VI (144). PDIs play an important role in viral infection *via* diverse mechanisms. PDIs were shown to be involved in viral entry of dengue virus by interaction with nonstructural protein 1 (145) and regulating integrin activity (146). PDIs bind capsid spike proteins of human astroviruses, the cause of gastroenteritis, thereby inhibiting viral entry, more precisely uncoating of the viral genome (147). PDIs regulate the redox state of hemagglutinin and neuraminidase and thereby virus attachment and influenza infection (148, 149). Also, in coronavirus disease 2019 (COVID-19), the spike glycoprotein of severe acute respiratory syndrome coronavirus type 2 (SARS-CoV-2) that binds to its receptor angiotensin-converting enzyme 2 contains two disulfide bonds (Cys379-Cys432 and Cys391-Cys525). To our knowledge, an interaction with PDI or Trx1 has so far not been determined; however, reducing agents showed antiviral properties, inhibiting viral entry (150).

## Conclusions

- The impact of extracellular thiol switches has long been underestimated.
- Extracellular thiol modifications of key proteins and receptors mediate i) signal transduction; ii) bacterial toxicity, adhesion, and invasion; and iii) viral invasion and infection.
- Extracellular thiol modifications regulate the adaptive immune response by modulating immunoglobulin antigen affinity and T-cell receptor epitope recognition. Whether this is an active mechanism of immune tampering or immune escape or even explains individual responses to pathogens remains to be elucidated.
- Members of the Trx family are involved in the modulation of thiol PTMs.
- Thiol modifications are novel targets for diagnosis and/or prognosis of inflammatory and infectious diseases and constitute potential therapeutic targets.

## Author Contributions

Conceptualization: KAB and E-MH. Writing, review, and editing: KAB, PLP, E-MH. All authors contributed to the article and approved the submitted version.

## Funding

This study was supported by the German Research Council (DFG: SPP1710) to E-MH (HA 8334/2-2). We acknowledge support by the Heinrich Heine University Düsseldorf.

## Conflict of Interest

The authors declare that the research was conducted in the absence of any commercial or financial relationships that could be construed as a potential conflict of interest.

## Publisher’s Note

All claims expressed in this article are solely those of the authors and do not necessarily represent those of their affiliated organizations, or those of the publisher, the editors and the reviewers. Any product that may be evaluated in this article, or claim that may be made by its manufacturer, is not guaranteed or endorsed by the publisher.
